# 4D-Flow MRI and Vector Ultrasound in the In-Vitro Evaluation of Surgical Aortic Heart Valves – a Pilot Study

**DOI:** 10.1007/s12265-024-10564-0

**Published:** 2024-10-04

**Authors:** Henrik Stephan, Linda Grefen, Dirk Clevert, Meike Onkes, Jin Ning, Nikolaus Thierfelder, Petra Mela, Christian Hagl, Adrian Curta, Maximilian Grab

**Affiliations:** 1 Department of Cardiac Surgery, LMU Hospital – Campus Großhadern, Marchioninistraße 15, 81377 Munich, Germany; 2 DZHK (German Centre for Cardiovascular Research), Partner Site Munich Heart Alliance, Munich, Germany; 3 Department of Radiology, LMU University Hospital, Munich, Germany; 4 Chair of Medical Materials and Implants, Department of Mechanical Engineering, TUM School of Engineering, and Design, Munich Institute of Biomedical Engineering, Technical University of Munich, Munich, Germany; 5 Siemens Healthineers AG, Erlangen, Germany

**Keywords:** 3D-Printing, 4D-MRI, Surgical Heart Valve, Vector Ultrasound

## Abstract

Introduction: The aim of this study was the initial investigation of 4D-Flow MRI and Vector Ultrasound as novel imaging techniques in the in-vitro analysis of hemodynamics in anatomical models. Specifically, by looking at the hemodynamic performance of state-of-the-art surgical heart valves in a 3D-printed aortic arch.

Methods: The mock circulatory loop simulated physiological, pulsatile flow. Two mechanical and three biological aortic valves prostheses were compared in a 3D-printed aortic arch. 4D magnetic resonance imaging and vector flow Doppler ultrasound served as imaging methods. Hemodynamic parameters such as wall shear stress, flow velocities and pressure gradients were analyzed.

Results: The flow analysis revealed characteristic flow-patterns in the 3D-printed aortic arch. The blood-flow in the arch presented complex patterns, including the formation of helixes and vortices. Higher proximal peak velocities and lower flow volumes were found for biological valves.

Conclusion: The mock circulatory loop in combination with modern radiological imaging provides a sufficient basis for the hemodynamic comparison of aortic valves.

**Graphical Abstract:**

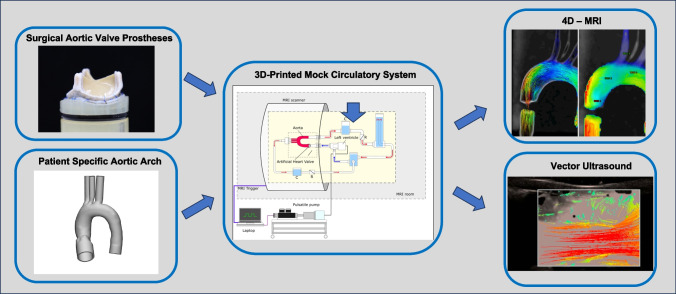

**Supplementary Information:**

The online version contains supplementary material available at 10.1007/s12265-024-10564-0.

## Introduction

Degenerative diseases of the aortic valve are one of the leading causes of cardiac morbidity and mortality [1]. From a very crude initial concept, the field of surgical aortic valves saved or enhanced the quality of life for millions of patients over the last decades. Due to the high demand for functional and lasting replacement options, the aortic valve device market turned into an ever-evolving field, trying to improve upon the existing bileaflet mechanical heart valves and xenogeneic bioprosthetic valves [2]. Additionally to these two established models, different options were developed over the years, ranging from trileaflet mechanical valves [3] to tissue engineered approaches [4].

One common aim for iterations of established valve systems and even radical innovations is to enhance the hemodynamic performance. Many factors, such as pressure gradients, velocities, flow patterns and thrombogenicity are inherently responsible for adequate blood flow and lasting functionality of the aortic valve and the overall cardiovascular system. The visualization and quantification of blood flow characteristics distal to the aortic valve have been at the center of cardiovascular research for decades [5]. In-vivo evaluation of patients has been performed using radiological modalities ranging from ultrasound (US) to magnetic resonance imaging (MRI) [6, 7]. In basic research, different technologies were developed over time, with particle image velocimetry (PIV) being one of the most applied techniques to visualize fluid characteristics in mock circulation setups [8].

In the past, these mock circulation setups mostly relied on acrylic vessels or silicone cast phantoms [9, 10]. The emergence of additive manufacturing opened new ways of creating accurate anatomical phantoms for integration in mock flow loops [11]. Besides the printing accuracy, an increasing range of printable materials allows for individualized design of the model’s properties, to more closely match the behavior of the human aorta. While these models offer great accuracy, current materials and printing techniques often result in printed vessel walls that are opaque, leading to limited usability in PIV measurements. This technical limitation makes exploring alternative imaging modalities necessary. Technological advances in the field of radiological imaging, offer new capturing techniques, such as 4D-Flow MRI, a type of three-dimensional, time resolved phase-contrast MRI [12]. This technology allows the visualization of disturbed flow patterns and quantification of flow parameters, such as velocity, pressure drops and WSS. In clinical research, 4D- Flow MRI has been widely used in the analysis of congenital heart defects [13], ventricular flow [14] and portal veins[15]. Besides 4D-Flow MRI, the computing power of modern sonographic imaging devices led to the introduction of vector flow doppler imaging, that allows the visualization of dynamic flow patterns, as well as the calculation of wall shear stress (WSS) and energy loss[16]. These imaging modalities give clinical radiology a broader toolbox to accurately examine patients. Furthermore, they can be used in translational and basic research for fast, non-invasive measurements. Therefore, the goal of this research project was the initial investigation of 4D-Flow MRI and Vector Ultrasound as novel imaging techniques in the in-vitro analysis of hemodynamics in anatomical models. Specifically, by looking at the hemodynamic performance of state-of-the-art surgical heart valves in a 3D-printed aortic arch.

## Methods

### Model Creation

**Fig. 1:**
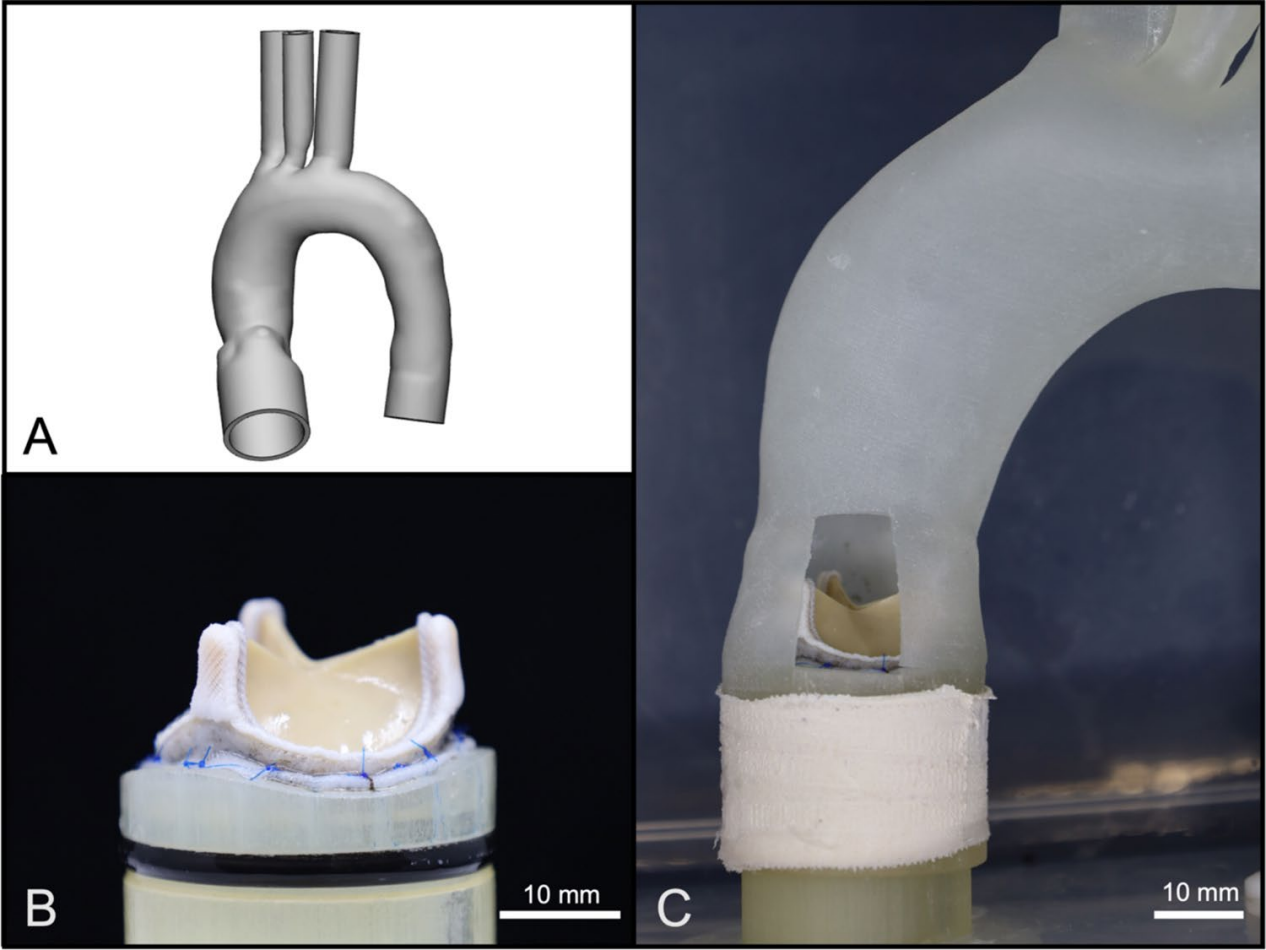
3D-Printed Arch and Valve Implantation; A: Digital model of the anatomical aortic arch with straightened connectors for improved implementation in the flow loop. B: Magna Ease biological valve sutured onto the customized valve holder. C: Valve placed inside the aortic arch with opening for visualization purposes

The main part of the flow loop setup is represented by a 3D-printed flexible thoracic aorta including the ascending aorta, the aortic arch and the descending aorta. The model creation workflow followed a previously published work [17]. Briefly, an anonymized contrast-enhanced CT dataset of a patient who had an indication for surgical aortic valve replacement with a 25 mm prosthesis, was segmented to extract the ascending aorta, aortic arch, aortic root and supra-aortic vessels. Different datasets were measured retrospectively to select a patient sized for a 25 mm aortic valve. Exclusion criteria were any of the following in the region of interest: poor image quality (i.e. device-related artefacts), pathologic diameter change, calcifications outside the aortic root and non-standard configuration of supra-aortic vessels. After segmentation of the blood volume, the digital model was hollowed by adding a constant wall thickness of 2.5 mm external to the blood volume [17]. All vessel ends were modified in a circular uniform diameter for easy attachment to standardized connectors (Fig. 1A). The proximal end of the left ventricular outflow tract was prolonged to allow for adequate sealing, as well as placement of the heart valve prostheses according to manufacturer’s specifications. Afterwards, the digital model was transferred into the slicing software Modeling Studio (Keyence Corp., Osaka, JP), subsequently uploaded onto a 3D-printer (Agilista 3200W, Keyence Corp.) and printed using a flexible, printing material (AR-G1L, Shore 35A, elongation at break: 160%, Keyence Corp.). After the printing process, the aortic phantom was taken from the build plate and soaked in boiling water to remove the water-soluble support material. Subsequently, the model was placed in a heating cabinet to dry for 24 h at 50 °C.

### Heart Valve Prostheses

To perform standardized comparative tests of different heart valve prostheses, a uniform prosthesis size of 25 mm (manufacturer’s specification) was selected for all valves tested in this study. Included are five different valves for surgical implantation, with two mechanical prosthetic valves (Masters Series 25, Abbott Laboratories, Chicago, USA; On-Xane-25, CryoLife Inc., Kennesaw, USA) and three different bioprosthetic heart valves (Epic 25 mm, Abbott Laboratories; Magna Ease 25 mm, Edwards Lifesciences Inc., Irvine, USA; Perimount 25 mm, Edwards Lifesciences Inc.). Individual valve mounts were designed to follow the individual curvature of the valve’s suture rings (Fig. 1B). Subsequently, valves were fixed to the mount using surgical sutures (Prolene 5–0, Ethicon Inc., Raritan, USA) and tested for paravalvular leakages. Each mount has a defined height, to allow for supra or intra-annular placement of the valves, according to manufacturer’s recommendations (Fig. 1C). The orientation of the mechanical valve leaflets was adjusted to match manufacturer’s recommendations. Bioprosthetic valves were stored in their original container with storage solution up until testing.

### Mock Circulation

**Fig. 2:**
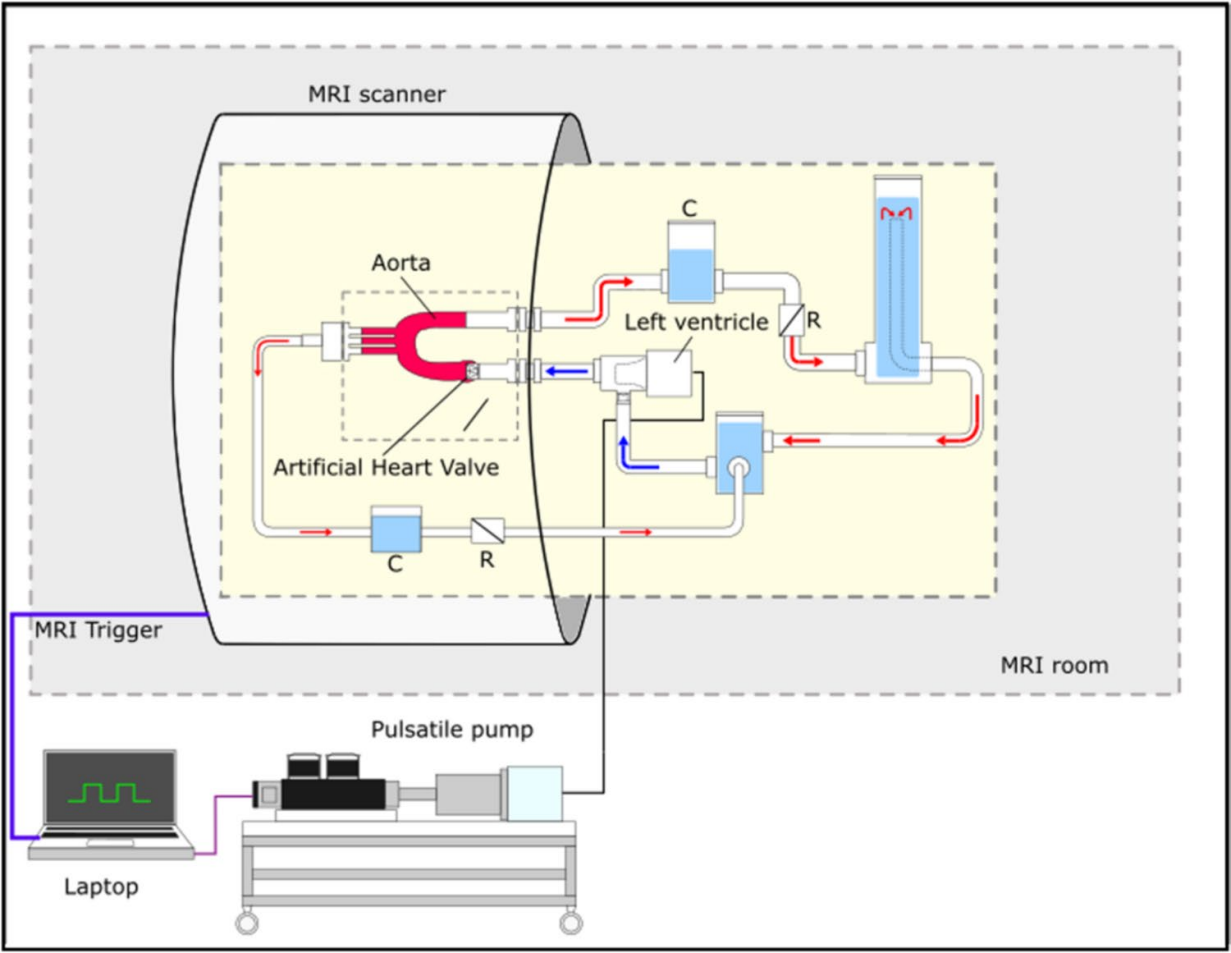
Schematic of the mock circulatory loop with an external pulsatile pump and MRI trigger. The inner loop consists of a newly developed ventricle, an airtight cylinder functioning as a compliance chamber (C), as well as several ball valves being utilized as resistance elements (R) to create physiological conditions

To allow for testing of the valves in an MRI setting, an entire MRI-compatible mock circulation setup was designed and constructed (Fig. 2). The setup was divided into two parts, the external drive unit and the internal fluid circulation unit. The external drive unit consisted of a dedicated computer, linear motor (PS01- 48 × 240 HP, NTI AG, Spreitenbach, CH) with corresponding driver (Series C1100, NTI AG). The linear motor was connected to a piston, which in turn is connected air-tight via a pneumatic hose to the fluid circulation unit. The connecting point also represents the heart of the mock circulation with a self-developed pump chamber, representing the left ventricle. To transfer the pneumatic force created by the piston to the test fluid, a rubber roll membrane with a defined volume of 80 ml was placed between the pneumatic and fluid chambers. The fluid chamber has a total volume of 100 ml resulting in a theoretical peak ejection fraction of 80%. An ejection fraction above physiological levels was chosen to adjust for the rigid nature of the artificial ventricle The chamber was connected to the valve mount via a straight rigid tube to allow for any flow disturbances to subside before passing through the valve prostheses. The 3D-printed aortic arch was then fixed to the valve mount which was placed in a plastic container. The container has five openings, for the proximal fluid entrance, the distal descending aorta and the supra-aortic vessels. After implantation of the valves in the aortic arch, the model was embedded in a hydrogel of 1% agar (Agarose, Sigma-Aldrich Corp., St. Louis, USA) to simulate the surrounding tissue and thereby reduce movement artefacts during MRI acquisition. Distal to the descending aorta and the supra-aortic vessels, a combination of compliance and resistance elements were placed to allow the approximation of the Windkessel-effect and peripheral vascular resistance. The compliance elements consist of an airtight cylinder filled partially with water and air, with a pneumatic valve at the top to adjust the height of the water column. The resistance element is realized through a ball valve that is placed distally to the compliance element. Therefore, realistic pressure conditions of 120/80 mmHg and a cardiac output of 4.6 l/min were achieved. Pressure was measured at the left ventricle, compliance chamber and descending aorta prior to MRI experiments. For all experiments, heart rate was set at 55 bpm, while systolic and diastolic pressure were adjusted to reach 120/80 mmHg. An ECG trigger signal was created and connected to the MRI according to manufacturer’s specifications. The trigger signal allowed the prospective synchronization of the ventricle movement with the acquisition time window. To simulate the viscous behavior of blood, a blood mimicking fluid (calculated viscosity 4.6cP) consisting of 40% glycerin (Rotipuran® ≥ 99.5%, Carl Roth GmbH, Karlsruhe, GER) and 60% distilled water was used [18].

### Radiological Imaging

Acquisition of the 4D-Flow MRI imaging was performed on a 1.5 T scanner (MAGNETOM Aera, Siemens Healthineers AG, Erlangen, GER) with an 18-channel body coil (Biomatrix Body 18, Siemens Healthineers AG) placed on top of the agar filled plastic box. The acquisition protocol consisted of a non-contrast-enhanced MR-angiography and the 4D-flow sequence. For 4D-flow an isotropic dataset with 25 phases and a slice thickness of 1.0 mm (TE 2.300, TR 38.800, FA 7°, matrix size: 298 × 298 px) was acquired. Velocity encoding was set at 150 cm/s for all measurements [19]. Evaluation and visualization of 4D-Flow MRI results was conducted using a dedicated radiological analysis software (cvi42, CCI Inc., Calgary, CA) [17]. Within the software, the blood volume was separated from surrounding motion artefacts. Four measurement planes were placed perpendicular to the vessel’s centerline, specifically proximal to the valve as a reference plane, 10 mm distal to the top of the valve, at the center of the ascending curvature and at the distal end of the aortic arch (Fig. 7). At each plane, velocity, tangential WSS and pressure drop with respect to the reference plane were measured. Calculation of WSS followed the publication by Stalder et al.[20]. It describes an interpolation of local velocity vectors along the contour of the underlying measuring plane. The effective orifice area (EOA) was calculated using the continuity equation (Eq. 1) with the velocity time integral in the left ventricular outflow tract (LVOT) and aortic valve (AV) derived from the underlying MRI dataset.

1 $$EOA= \frac{{d}_{LVOT}^{2}* \frac{\pi }{4}*{VTI}_{LVOT}}{{VTI}_{AV}}$$

Equation 1: Continuity equation to determine the EOA; d = diameter; VTI = velocity time integral.

Sonographic imaging was performed using a dedicated sonography device (Resona 9, Mindray Medical Int. Ltd., Shenzhen, CN) and the v-flow protocol, developed for carotid artery imaging. For image acquisition a linear array transducer (L14-3WU, Mindray Medical Int. Ltd.) was placed on to the agar block in correspondence to the above-mentioned planes, placing the center of the transducer on the according plane. The acquisition window was increased to the biggest possible size (20 × 30 mm) while all other parameters were set to the most precise setting available (acquisition time: 2 s; acquisition quality: 7). Since the acquisition window was developed for application at the carotid bifurcation, measurements had to be split into two parts at the inner and outer curvature of the aorta to cover the entire cross-section, due to the smaller ROI of the acquisition window. Flow velocity, total WSS at five spots along the aortic wall as well as the oscillatory shear index (OSI) were calculated from the measurements. The OSI was calculated as an expression for the magnitude and change in direction of local WSS described by the following formula:

2 $$OSI= \frac{1}{2}*(1.0- \frac{AWSSV}{AWSS})$$

where AWSSV = magnitude of the time-averaged WSS vector, and AWSS = time-averaged WSS magnitude [21].

## Results

### MRI Image Analysis

**Fig. 3:**
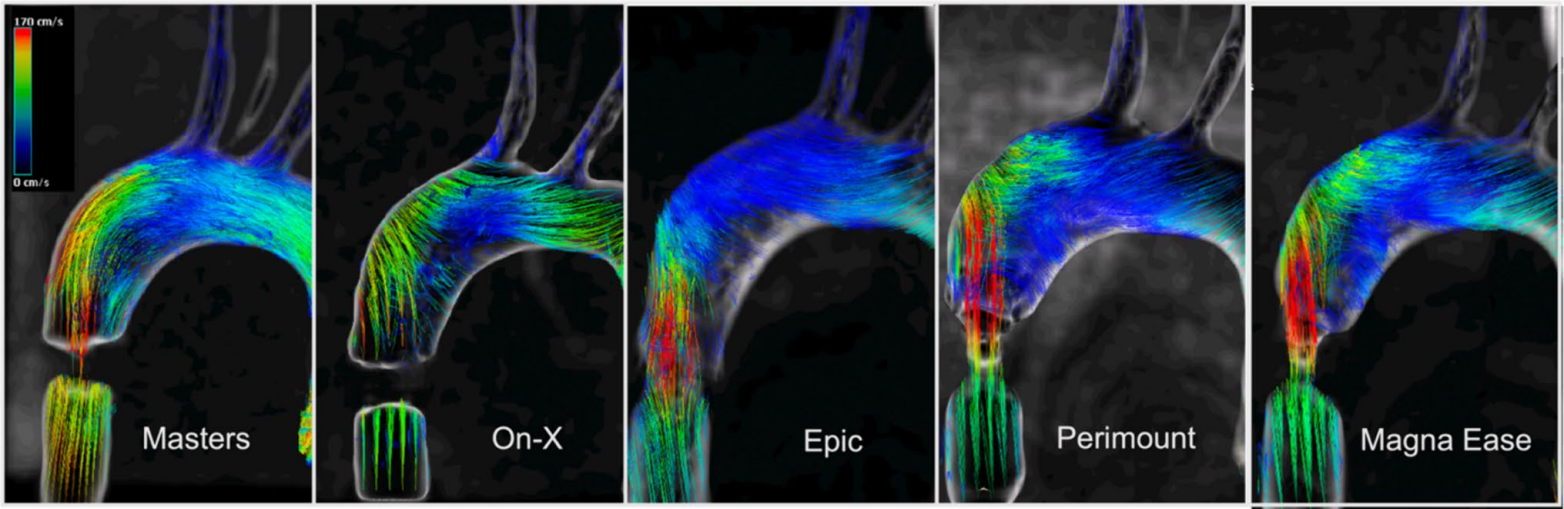
4D-MRI pathline visualization of the peak flow velocity in the aortic arch for all surgical aortic valves during peak systole; Mechanical valves showed artefacts around the valvular plane, leading to signal loss

Visualization of flow patterns and pathlines was achieved in the aortic arch, the brachiocephalic trunk and the left subclavian artery (Fig. 3). Visualization in the left common carotid artery proved difficult due to the smaller diameter of the vessel and was not achieved for all datasets.

For the Masters mechanical valve, pathline visualization revealed a central jet during peak systole that closely followed the outer curvature of the ascending aorta. This led to a decentralized flow pattern with lower velocities along the inner curvature. During peak systole, recirculation zones with the formation of sinus vortices at both sides of the proximal aortic root were visible. WSS analysis revealed high local load on the outer curvature of the ascending aorta during peak systole, closely following the high velocity. Other parts of the aortic arch showed no increase in WSS during the systolic phase.

**Fig. 4:**
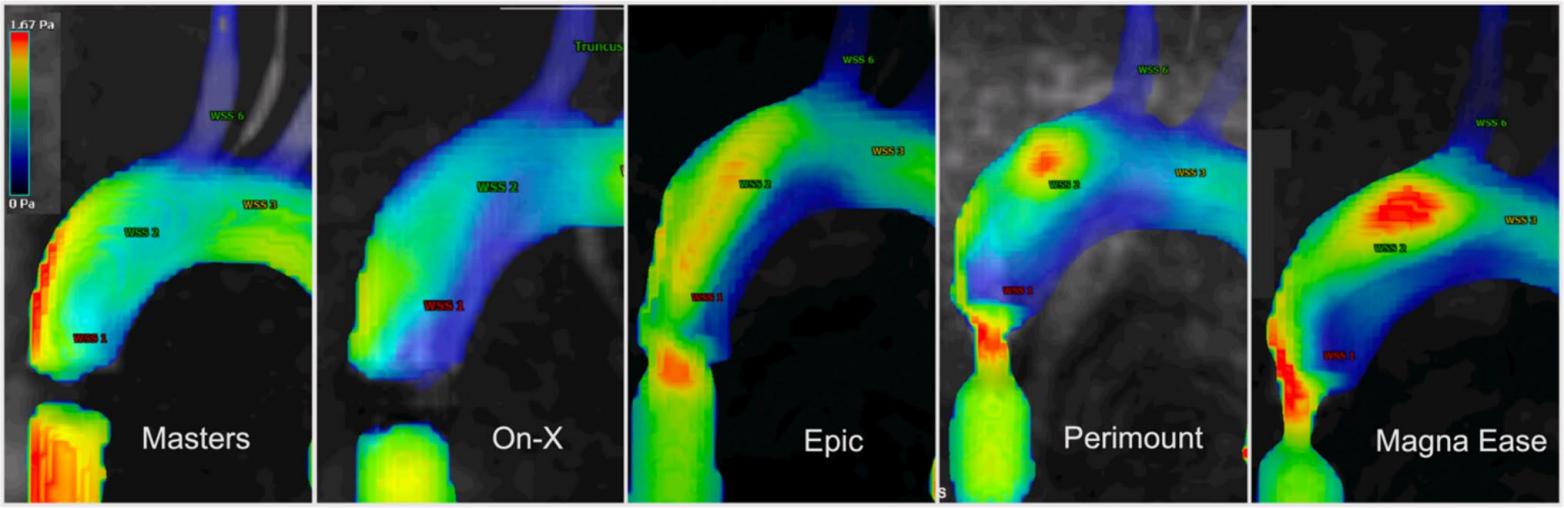
4D-MRI wall shear stress visualization for all surgical valves during peak systole; Mechanical valves showed artefacts around the valvular plane, due to the valve material

The On-Xane mechanical valve showed a slightly less centralized jet during peak systole. This led to a more even distribution of flow velocity across the aortic diameter, while still showing a tendency towards higher flow velocities along the outer curvature. This even distribution could also be visualized in the WSS analysis, where a moderate load and distribution across the ascending aorta could be observed (Fig. 4).

The examination of the porcine bioprosthetic valve Epic showed a high velocity central jet hitting the outer curvature of the ascending aorta and partially reflecting onto the top of the inner curve. The central jet also showed a symmetric distribution with a tendency of tilting towards the outer curvature, resulting in an asymmetric distribution of systolic flow. WSS analysis revealed a high load on the outer curvature with an added high stress put on the anterior ascending aorta, close to the trunk.

The Perimount bioprosthetic valve showed a central jet with high symmetric velocity, reflecting from the outer curvature of the ascending aorta. Visualization of WSS was consistent with the other bioprosthetic valves, where a high WSS occurred on the anterior wall of the ascending aorta.

Lastly, the strong central jet could also be observed in the latest generation of bovine bioprosthetic valves, the Magna Ease. Here, the jet also showed a central symmetric velocity distribution distal to the valve followed by a tendency to adhere to the outer curvature, leading to asymmetric flow distribution. Due to the sharp angulation of the jet, wall shear stress was increased on the outer curvature close to the aortic root (Fig. 4). Similarly, wall shear stress was also increased on the anterior side of the ascending curvature.

### MRI Quantitative Analysis

**Fig. 5:**
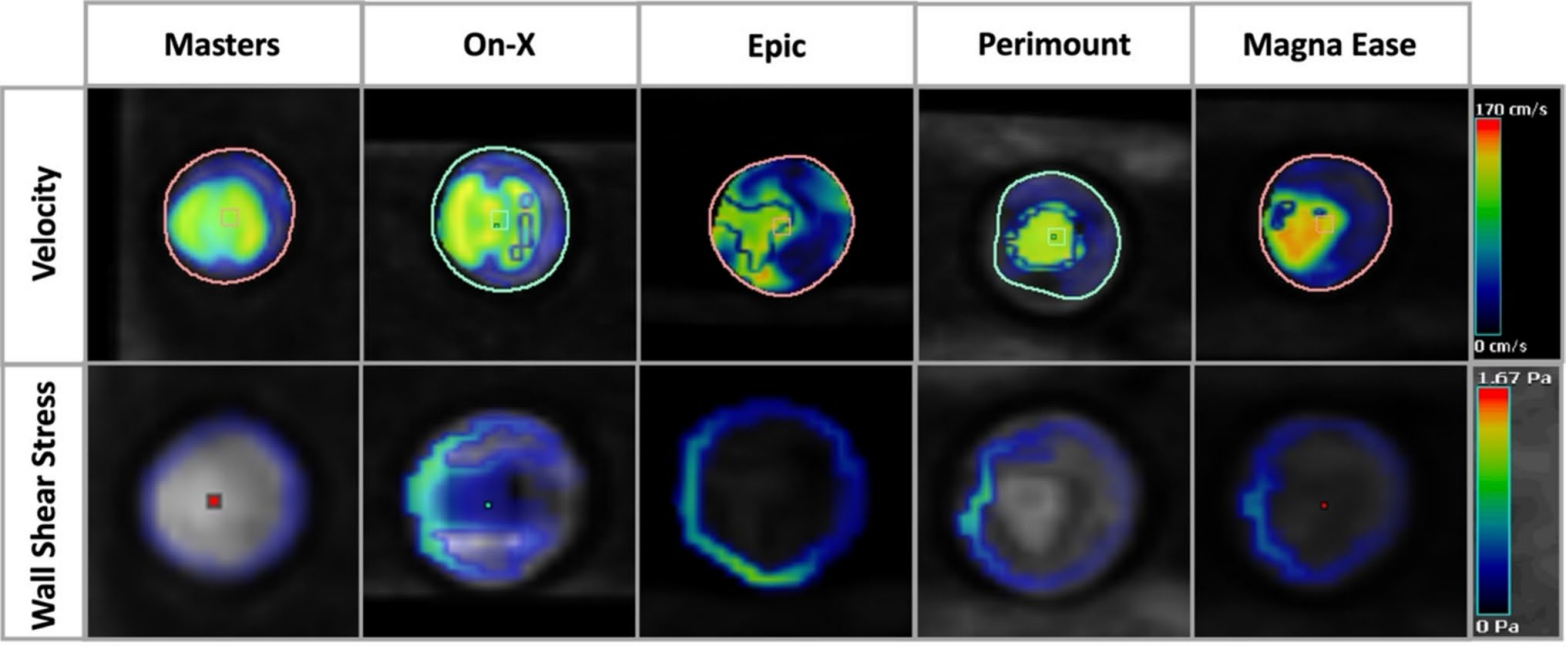
Cross-sectional view of the valvular plane for all tested artificial heart valves. First row present the flow velocity during peak systole; second row presents the wall shear stress during peak systole

**Fig. 6:**
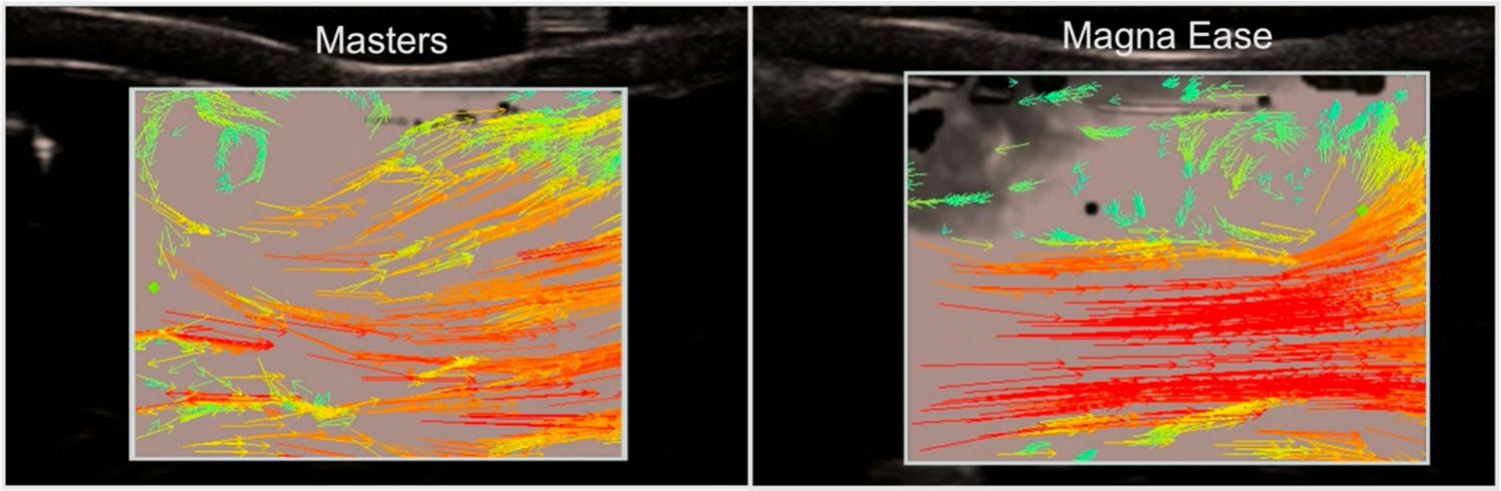
Vector ultrasound of the aortic root for a mechanical (left) and biological (right) valve during peak systole; The measurement plane corresponds to plane 1 from Fig. 7; In both images the vortices at the upper wall are prominently visible with a main difference being the strongly developed central jet in the biological valve, while the jet for the mechanical valve appears more diffuse

**Fig. 7:**
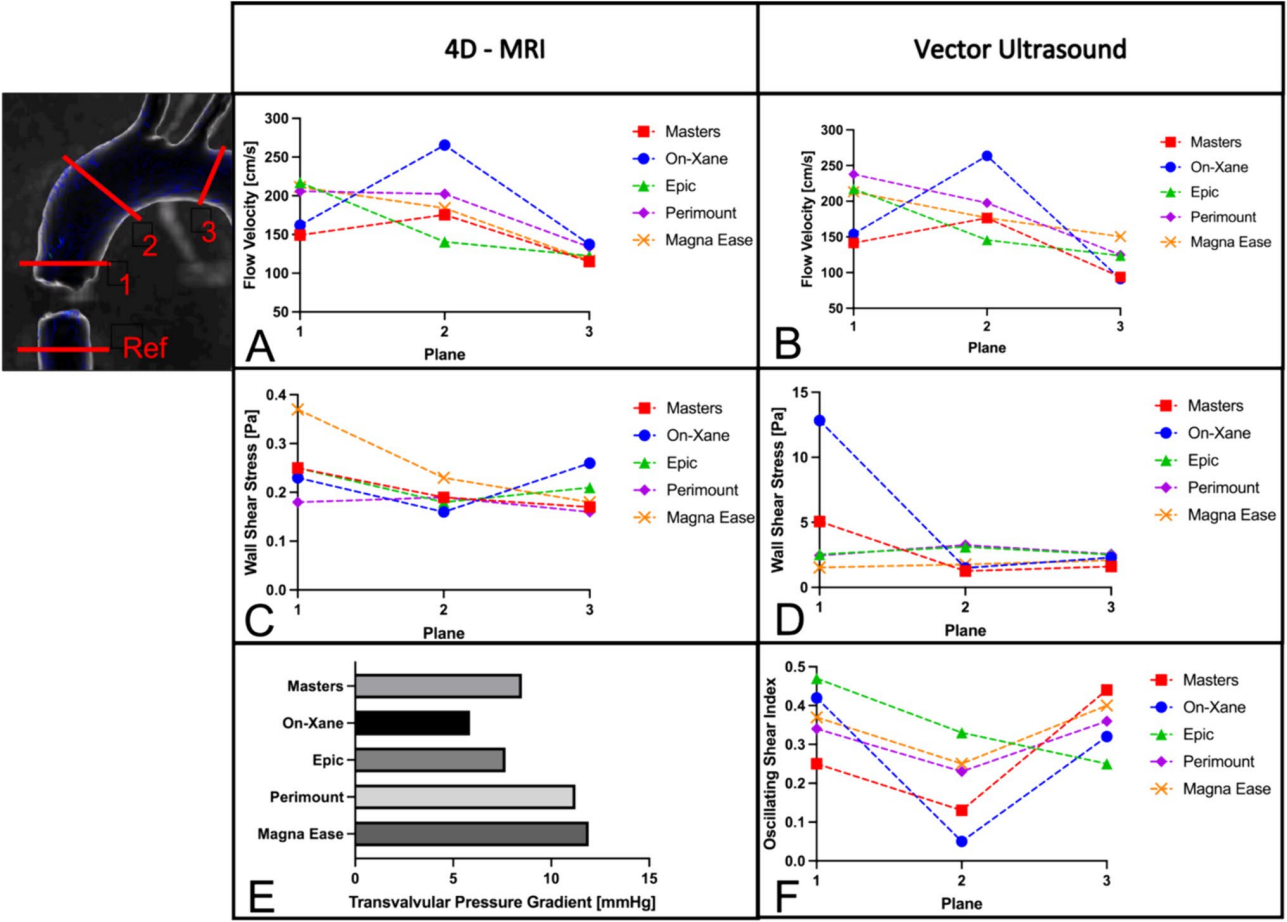
Quantification of 4D-MRI and Vector ultrasound; A: Peak velocity in every plane in 4D-MRI. B: Peak velocity in every ROI in Vector ultrasound. C: 4D-MRI Wall Shear Stress. D: Vector ultrasound Wall Shear Stress. E: Transvalvular Pressure Gradient measured in 4D-MRI. F: Oscillating Shear Index calculated in Vector ultrasound. Measuring planes can be seen in the schematic to the left. The center of the ultrasonic transducer was placed at the respective measuring plane

Cross-sectional visualization of flow velocities and WSS for all valves can be seen in Fig. 5. Measured velocity values in the three ROI planes are shown in Fig. 6A. While all biological heart valves show a constant decrease in peak velocity between the planes, both mechanical heart valves cause an increase in peak velocity, reaching the highest value in the ascending aorta (Plane 2). In this study, the On-Xane mechanical valve reached the highest overall peak velocity of 265,6 cm/s in the ascending aorta while the Epic bioprosthetic valve exhibits the slowest velocity in the ascending aorta of 140.5 cm/s (Fig. 7A). Analysis of the tangential WSS presented the highest WSS closer to the aortic bulbus (Fig. 7, measuring plane 1) with a steady drop towards the descending aorta. The overall highest WSS could be observed for the Magna Ease biological valve at the aortic root, reaching 0.37 Pa (Fig. 7C). Peak pressure gradient measurement of mechanical valves between the proximal inlet and the aortic root revealed a gradient of 5.86 mmHg for the On-Xane valve and 8.50 mmHg for the Masters valve. The biological valves reached a peak pressure gradient of 7.67 mmHg for the Epic, 11.24 mmHg for the Perimount and 11.91 mmHg for the Magna Ease (Fig. 7E). Additionally, EOA was measured inside the respective valve, with On-X (2.8 cm^2^) and Masters (2.1 cm^2^) having the largest EOA, followed by the biological heart valves, Epic (2.0 cm^2^), Perimount (1.4 cm^2^) and Magna Ease (1.3 cm^2^). Especially, the Perimount and Magna Ease valve showed artifacts around the valve mount, which led to some difficulties when setting the plane for velocity assessment in the LVOT.

### Sonographic Image Analysis

Vector flow analysis of the surgical valves revealed overall strong signal during the systolic phase, while diastolic phase led to many visible artefacts (Video File in Supplement). In the aortic bulbus, the mechanical valves revealed a central jet, showcasing the disturbance created by the two semicircular leaflets (Fig. 6). Both Masters and On-Xane mechanical valves displayed large recirculation areas and the distinct formation of vortices close to the coronary arteries. In the ascending aorta, as well as the descending aorta, flow patterns exhibited uniform flow with no distinct recirculation areas. The Epic bioprosthetic valve showed a broader central jet during peak systole with a distinct recirculation area above the aortic annular plane. For the Perimount valve, a broader central jet could be observed during peak systole, leading to a smaller low-flow area at the aortic wall. This also significantly reduced the occurrence of turbulences and recirculation. A similar behavior could be observed in the aortic root proximal to the Magna Ease valve, with a large recirculating turbulence next to the central jet (Fig. 6). Similarly to the mechanical valves, the flow pattern in the ascending and descending aorta revealed a uniform flow with small recirculation areas for all biological valves.

### Sonographic Quantitative Analysis

Analysis of peak flow velocity during systole revealed a similar behavior to the MRI analysis with mechanical valves showing a lower flow velocity in the aortic bulb, an increase in the ascending aorta, followed by a decrease in the descending aorta (Fig. 7B). Biological valves created the highest peak flow velocity in the aortic root with a steady decrease along the aortic arch. The highest overall velocity in the sonographic imaging was measured for the On-Xane valve at 263.6 cm/s. Biological valves displayed slightly lower peak velocities with the Perimount valve reaching the highest value of 237.9 cm/s directly in the aortic root. WSS measurements along the aortic wall also exposed big differences between mechanical and biological valves. The Masters valve (5.07 Pa) and the On-Xane valve (12.83 Pa) exhibited much higher total WSS in the aortic root compared to the biological valves (Epic: 2.55 Pa; Perimount: 2.46 Pa; Magna Ease: 1.53 Pa, Fig. 7D). In the ascending aorta, the WSS dropped for the mechanical valves and increased for the Epic and Perimount valve, with all valves reaching similar wall shear stress values in the descending aorta. The OSI as a measure for the change in direction and magnitude of WSS, is visualized in Fig. 7F. Mechanical valves reveal a higher initial OSI in the aortic root, with a drop in the ascending aorta. Biological valves showed a lower rate of change compared to the mechanical valves, with a slight drop of the OSI in the ascending aortic arch.

## Discussion

The introduction of additive manufacturing in the medical field enabled for the creation of highly accurate anatomical models based on underlying radiological data. This study focused on the application of the 3D-printing technology to create a flexible aortic arch for testing of the hemodynamics caused by the implantation of different surgical aortic valves. So far, the hemodynamic evaluation of such valves has been limited to PIV measurements using pulse duplicators[22]. The advancements in computing power seen in the last decades accelerated the use of computational fluid dynamics, as well as 4D-Flow MRI to further investigate hemodynamics in the aorta [23, 24].

4D-Flow MRI has proven to be a vital tool in clinical assessment with broad opportunities for further validation in an in-vitro setting [19, 25]. It allows for a holistic examination of the cardiovascular region of interest, opening new possibilities in the diagnosis and prevention of i.e., aortic aneurysms. The larger region of interest is especially beneficial when comparing the technology to PIV, where the camera only allows for a limited field of view.

The measurement of WSS in the entire aortic arch is a clear benefit of the 4D-Flow MRI with numerous applications in both basic research and clinical routine. The WSS values measured in our 3D model show great comparability to the WSS values measured in patients by Bürk et al., who looked at the WSS in healthy and dilated aortas [26]. While additional comparative studies are required, this shows a good initial approximation of the WSS values created by the flow loop to patient-based data.

This slight discrepancy in WSS between our model and the values measured in patients can be explained by the mechanical properties of the 3D-printed aortic arch. Current 3D-printed flexible models lack the possibility to add fiber-orientation and therefore are not able to mimic the exact native aorta’s non-linear elastic behavior. [17] Another explanation for this mismatch could be the material-geometry coupling of aortic replicas described by Comunale et al., which confirms, that not only material properties, but also geometry have an impact on the hemodynamic parameters [27].

Besides the quantification of WSS, the localization of higher WSS areas is important to predict the risk of aortic aneurysm formation [28]. The increase in both WSS and OSI has been associated with an upregulation of inflammatory markers [29]. Especially, the localization of increased WSS on the anterior wall of the ascending aortic arch for all biological valves is a key finding of our study. This has been previously described by Farag et al., for patients undergoing transcatheter aortic valve replacement with a Sapien 3 transcatheter valve, where a large percentage of patients displayed an increase in WSS on the anterior wall compared to a control group [30]. The increased WSS on the outer curvature of the ascending aortic arch observed is in accordance to previously described findings by in-vitro PIV studies [31].

Another parameter measured via 4D-Flow MRI was the pressure drop across the artificial heart valves. The pressure gradient helps in the evaluation of the overall performance of native and artificial valves and is a standard parameter in the sonographic assessment of patients. For the mechanical valves, Hatoum et al. measured the pressure gradients for both the On-Xane as well as the SJM Masters valve reaching 4.15 and 4.75 mmHg in their in-vitro setting, respectively [32]. Lee et al. analyzed the performance of Magna Ease bioprosthetic valves in patients who underwent surgical aortic valve replacement, where the mean pressure gradient for the 25 mm valve reached 12.2 mmHg [33]. Compared to these studies, the pressure gradient for the mechanical valves was a bit lower in our study.

While the pressure drop is a valuable metric in determining the performance of an artificial heart valve, comparison between in-vitro and in-vivo studies can prove challenging. A multitude of variables can have an impact on the measured pressure drop, ranging from the exact position of the measurement, the aortic diameter, measurement technique, prosthesis size and additionally, fluid viscosity in case of in-vitro studies. Pressure drops are therefore most comparable within the same experimental setup and a comparison to the aforementioned studies can only be seen as informative.

The EOA of surgical valves is another factor having an influence on the transvalvular pressure and flow velocity. Different surgical valves with the same size (e.g., 25 mm) can have highly varying EOA. Pibarot et al. have determined the EOA of different surgical valve models and sizes for comparison, with the 25 mm Edwards Perimount having an EOA of 1.8 ± 0.4 cm^2^ while the 25 mm On-X has a EOA of 2.4 ± 0.8 cm^2^ [34]. This corresponds to a difference in EOA of 33%, highlighting the importance of individualized prosthesis selection for every patient. The effect of the increased EOA can also be observed in our study, since the mechanical valves show a lower transvalvular pressure gradient compared to the biological valves.

The usage of vector sonography is a rather young technique with great potential to improve treatment of cardiovascular patients. The current clinical use-case of quantifying the WSS in carotid arteries is a first step of improving one of the most commonly used radiological modalities [35]. Its application in a benchtop setting offers great opportunities to analyze anatomical structures, which are not easily accessible in a clinical setting. Compared to the 4D-Flow MRI, vector ultrasound allows a much closer analysis of small cardiovascular structures and flow phenomena, like vortices at the aortic valve. In this study, motion artefacts were presented, especially during the diastolic phase, which might be caused by the reflective nature of the 3D-printed material. During the systolic phase, no artifacts were visible, allowing for a precise analysis of the flow conditions in the ROI. The observed vortices for both the mechanical and bioprosthetic valves match closely to the previously described hemodynamics caused by the different designs [36]. The mechanical valves show three distinct forward jets with small recirculation zones distal to the valvular plane, while the bioprosthetic valves display one larger central forward jet with counter-rotating recirculation areas surrounding the central jet. The design improvements from the Perimount to the Magna Ease valve could be partially confirmed in the quantitative analysis. The Magna Ease, which is designed to have a smaller sewing ring and therefore larger EOA has lower WSS in the aortic root, whereas the Perimount valve shows a slightly higher velocity at the aortic arch.

The biggest difference in the parameters derived from MRI and ultrasound is the WSS, especially in the aortic root. Vector ultrasound presents consistently higher WSS values, reaching a tenfold higher value for the On-X mechanical valve. These WSS values are much closer to values derived from CFD analyses of the aortic arch [37, 38]. The difference could also be explained by the different measurement techniques employed by MRI and vector ultrasound. MRI using an interpolation of velocity vectors along a circumferential contour, while vector ultrasound uses singular points in the longitudinal axis of the aortic wall.

## Limitations

While this study presents in-vitro results that are comparable to clinical data, there are still a few limitations to the setting. Firstly, the flexible material used for the anatomical aortic arch does not offer the same mechanical properties as a native human aorta. The fixed wall thickness and linear elastic behavior of the material are clear limitations. Furthermore, as described in the discussion, the geometry of the arch has an additional impact on the hemodynamic parameters. To minimize the effects of these three aspects, we decided to use the same arch design for all valves to properly compare them, nevertheless, this is an aspect that has to be taken into account when evaluating the collected data. Additionally, the presented model is lacking coronary perfusion. Due to the mechanical properties of the printing material, inclusion of coronary vessels would have led to an unnatural enlargement of the aortic root, which was previously described by other research groups as well. Secondly, although vector ultrasound presents a promising technique to analyze hemodynamic effects in the cardiovascular system, the technique is still rather new and requires further improvements to become a staple in the clinical field. Especially the limited depth of the ROI window represents a limitation when analyzing the aorta, since there is no possibility to examine the entire cross-section at once. Finally, this pilot-study lacks the comparison to measurements in a real-life patient, which is a clear limitation.

## Conclusion

Combining novel radiological imaging modalities with 3D-printed anatomical models offer great possibilities to further improve the in-vitro analysis of the hemodynamic effects of medical implants. This will be a valuable addition to a more patient-oriented medicine that can prevent patient-prosthesis mismatch and reduce overall complication rate through the usage of patient-specific anatomies in the mock circulatory loop. This study presents a first pilot study, which will lead to further research projects, focusing on the analysis of other cardiovascular implants, as well as the impact of specific anatomical configurations on the hemodynamic.

## Supplementary Information

Below is the link to the electronic supplementary material.

Supplementary file1 4D-MRI Visualization of Flow Pathlines in a 3D printed aortic arch with a Magna Ease valve implanted (MP4 1459 KB)

Supplementary file2 Vector Ultrasound Visualization of the Aortic Bulbus right after a Magna Ease biological heart valve (MP4 12599 KB)

Supplementary file3 (MP4 863 KB)

Supplementary file4 (MP4 12619 KB)

Supplementary file5 (JPG 904 KB)

Supplementary file6 (PNG 90 KB)

## Data Availability

The data that support the findings of this study are available from the corresponding author upon reasonable request.

## Declarations

### Statement on Human Studies

No human studies were carried out by the authors for this article.
