# Supplementary figures and images for: 4D-Flow MRI and Vector Ultrasound in the In-Vitro Evaluation of Surgical Aortic Heart Valves – a Pilot Study
Source: J Cardiovasc Transl Res. 2024 Oct 4;18(1):158–68. doi: 10.1007/s12265-024-10564-0 (PMC11885334; doi:10.1007/s12265-024-10564-0)

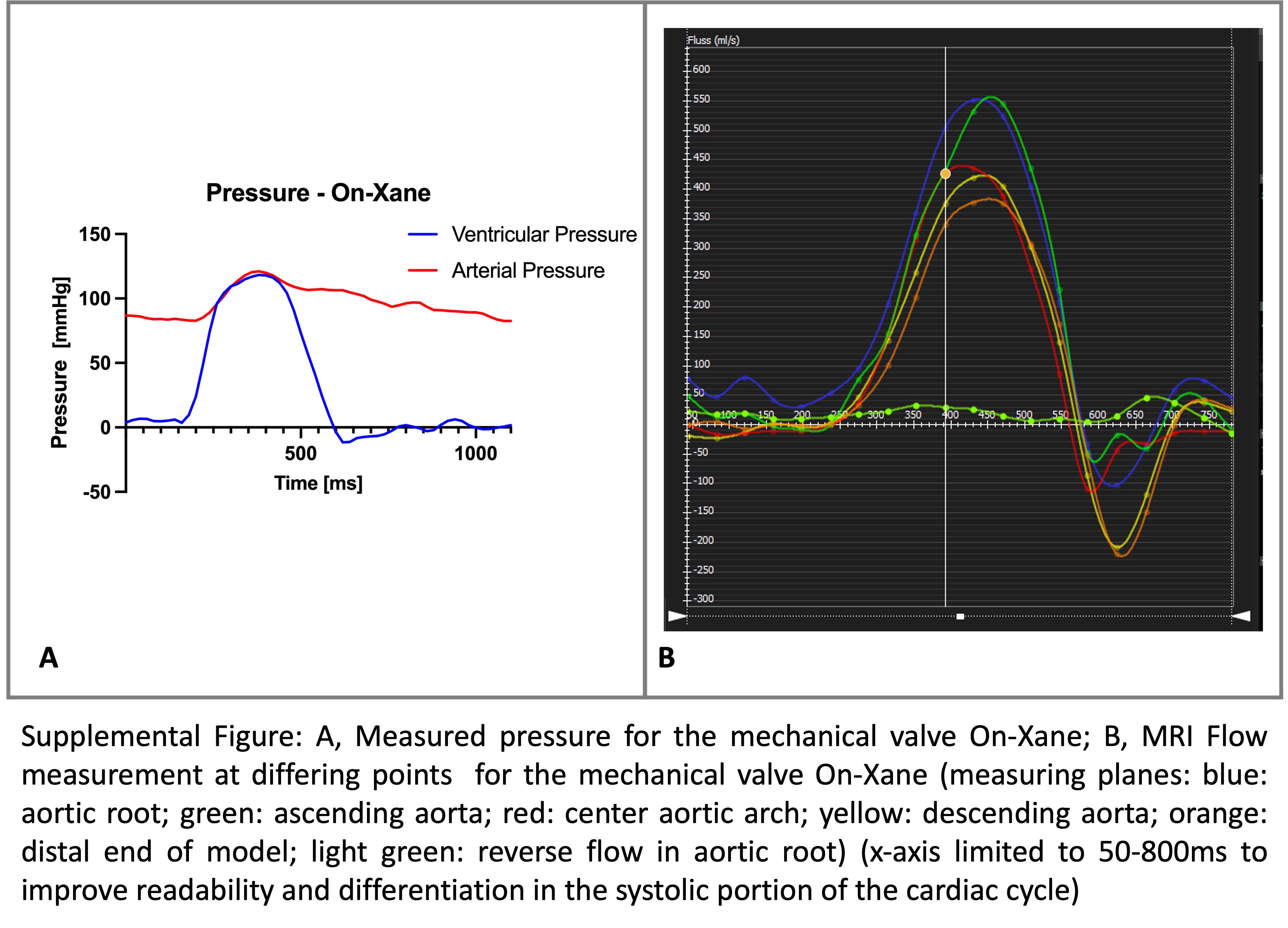

Supplement: Supplementary file 5 — Supplementary file5 (JPG 904 KB) [file 12265_2024_10564_MOESM5_ESM.jpg]

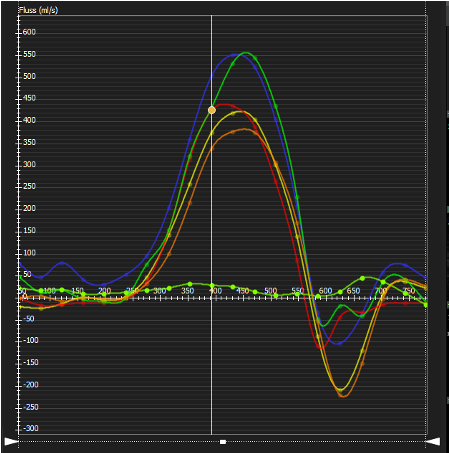

Supplement: Supplementary file 6 — Supplementary file6 (PNG 90 KB) [file 12265_2024_10564_MOESM6_ESM.png]
